# A Virtual Comparison of the eCLIPs Device and Conventional Flow-Diverters as Treatment for Cerebral Bifurcation Aneurysms

**DOI:** 10.1007/s13239-019-00424-3

**Published:** 2019-07-08

**Authors:** T. W. Peach, D. Ricci, Y. Ventikos

**Affiliations:** 1 Department of Mechanical Engineering, University College London, Torrington Place, London, WC1E 7JE UK; 2 eVasc Neurovascular, 107-1099 8th Avenue West, Vancouver, BC V6H 1C3 Canada; 3 Division of Cardiology, University of British Columbia, Vancouver, BC Canada

**Keywords:** Cerebral aneurysm, Bifurcation aneurysm, Medical device, Flow-diverter, Stent

## Abstract

**Purpose:**

Effective, consistent, and complication-free treatment of cerebral bifurcation aneurysms remains elusive despite a pressing need, with the majority of lesions presenting in such locations. Current treatment options focus either on aneurysm coil retention, supported by a stent-like device positioned in the parent vessel lumen, or intrasaccular devices that disrupt flow within the aneurysm dome. A third alternative, i.e., the use of conventional (intraluminal) flow-diverters to treat such bifurcation aneurysms raises the problem that at least one daughter vessel needs to be jailed in such a deployment. The eCLIPs is a stent-like device that offers the possibility of flow-diversion at the aneurysm neck, without the drawbacks of daughter vessel occlusion or those of intrasaccular deployment.

**Methods:**

In this study the eCLIPs device was virtually deployed in five cerebral bifurcation aneurysms and compared with a conventional tubular flow-diverter device. Computational fluid dynamics (CFD) simulations of the aneurysm haemodynamic environment pre- and post-implantation were conducted, and focussed on metrics associated with successful aneurysm occlusion. Absolute and relative reductions in aneurysm inflow rate (Q) and time-averaged wall shear stress (TAWSS) were recorded.

**Results:**

The eCLIPs device was found to perform in a similar qualitative fashion to tubular flow-diverters, with overall reduction of metrics being somewhat more modest however, when compared to such devices. Aneurysm inflow reduction and TAWSS reduction were typically 10–20% lower for the eCLIPs, when compared to a generic flow diverter (FD_BRAIDED_) similar to devices currently in clinical use. The eCLIPs was less effective at diffusing inflow jets and at reducing the overall velocity of the flow, when compared to these devices. This result is likely due to the larger device pore size in the eCLIPs. Notably, it was found that the eCLIPs provided approximately equal resistance to flow entering and exiting the aneurysm, which was not true for the FD_BRAIDED_ device, where high-speed concentrations of outflow were seen at the aneurysm neck along with local TAWSS elevation. The clinical implications of such behaviour are not examined in detail here but could be significant.

**Conclusions:**

Our findings indicate that the eCLIPs device acts as a flow-diverter for bifurcation aneurysms, with somewhat diminished occlusion properties comparing to tubular flow diverters but without the jailing and diminished flow evident in a daughter vessel associated with use of conventional devices.

## Introduction

The majority of cerebral aneurysms are known to occur at vessel bifurcations.^2^,^6^ Despite significant innovation in cerebral aneurysm treatment in the past two decades, only a small number of dedicated devices are available to specifically treat bifurcation aneurysms. Even fewer options are available when more challenging wide-necked aneurysms must be treated. The majority of these devices act as supports to enable aneurysm coiling, such as the pCONus and pCANvas devices (Phenox, Bochum, Germany) and PulseRider (Pulsar Vascular/CERENOVUS J&J, Irvine, CA, USA), while a smaller number of devices act as intrasaccular flow disruptors like the WEB (Sequent Medical/MicroVention Terumo, Aliso Viejo, CA, USA), Luna/Artisse (Medtronic, Dublin, Ireland) and the Medina (Medtronic/Covidien/eV3, Dublin, Ireland) devices.

There are well-documented positive outcomes from sidewall aneurysms treated with conventional, tube-like, braided flow-diverter devices, including the SILK/SILK + (Balt Extrusion, Montmorency, France), Pipeline Embolization Device (PED) (Medtronic/Covidien, Dublin, Ireland), Flow Re-direction Endoluminal Device (FRED) (MicroVention Terumo, Aliso Viejo, CA, USA) and Surpass (Stryker Neurovascular, Fremont, CA, USA).^5^ The use of conventional flow-diverters in bifurcation cases, however, remains controversial due to the necessity of jailing a daughter vessel with the low-porosity device mesh. A number of studies have reported daughter vessel occlusion or incomplete aneurysm obliteration with neck remnants in such cases.^1^,^9^,^15^,^26^,^31^,^32^,^37^ Although vessel occlusion is often asymptomatic, given sufficient collateral flow in the Circle of Willis,^1^ the long term risks and reduction in retreatment options have made such off-label use of conventional flow diverters in bifurcation aneurysms a rarity.

All bifurcation-specific flow-diverter devices currently in clinical use (WEB, Artisse and Medina) require the device to be inserted into the aneurysm dome. As such, while these devices can cover a range of aneurysm sizes, their use is typically limited to aneurysms where there is good compliance between the aneurysm and device shape. Consequently the success of treatment is highly dependent upon accurate 3D measurements of the aneurysm geometry.^25^ Additionally, reports in the literature point towards concerns over compaction of intrasaccular flow-diverter devices and poor outcomes when deployed in partially thrombosed lesions.^3^,^4^

**Figure 1:**
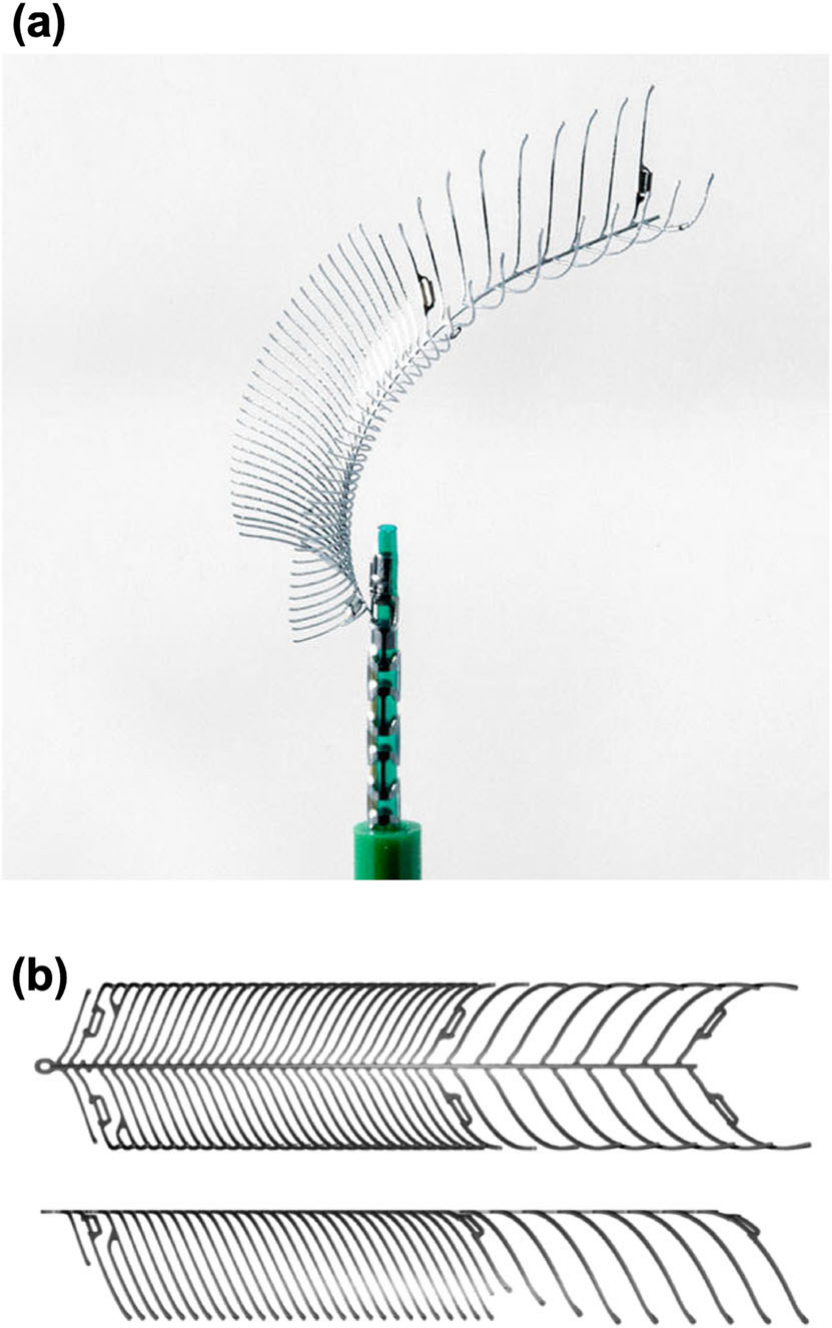
The eCLIPs device (a) as packaged and deployed *via* microcatheter and (b) in top and side view during manufacture.

The eCLIPs (Evasc Medical Systems Corp.) is a stent-like device that can be deployed to cover the neck region of a bifurcation aneurysm, reducing inflow, while still allowing unfettered access to daughter vessels and microcatheter access to the aneurysm dome for adjunctive coil placement. The eCLIPs—shown in Fig. 1—is a doubly-curved device with two distinct sections: one section with a set of anchoring ribs (visible in the distal portion of the device shown in the figure) which secure the device in a daughter vessel lumen, and a second section with higher-density ribs that cover the aneurysm neck, which are capable of both retaining coils and diverting flow. The device is non-cylindrical, resulting in greater compression/expansion at the vessel wall and giving good apposition over a range of vessel sizes. The porosity of the eCLIPs, approximately 65% with a range of 58–77%, is similar to that of a conventional flow diverter. Complete details of the eCLIPs design, delivery *in vivo*, and corresponding antiplatelet regimen are available in the literature.^8^,^17^,^28^

Use of the eCLIPs pre-clinically and clinically as a coil-assist device is documented in the literature.^8^,^17^,^28^ In two clinical cases the device was used without adjunctive coiling—acting exclusively as a flow-diverter device.^8^ The successful isolation of the lesion in these cases raises the possibility of using the eCLIPs as a dedicated flow-diverter in other aneurysm geometries.

In this study the flow-diverting effect alone of the eCLIPs is investigated to ascertain whether treatment without coil placement would be effective in five bifurcation aneurysm cases. The eCLIPs is virtually compared to a conventional flow-diverter device deployed, with both the absolute value and relative reduction in aneurysm inflow and wall shear stress considered as metrics of treatment success.

## Methodology

### Aneurysm Geometries and Virtual Deployment

**Figure 2:**
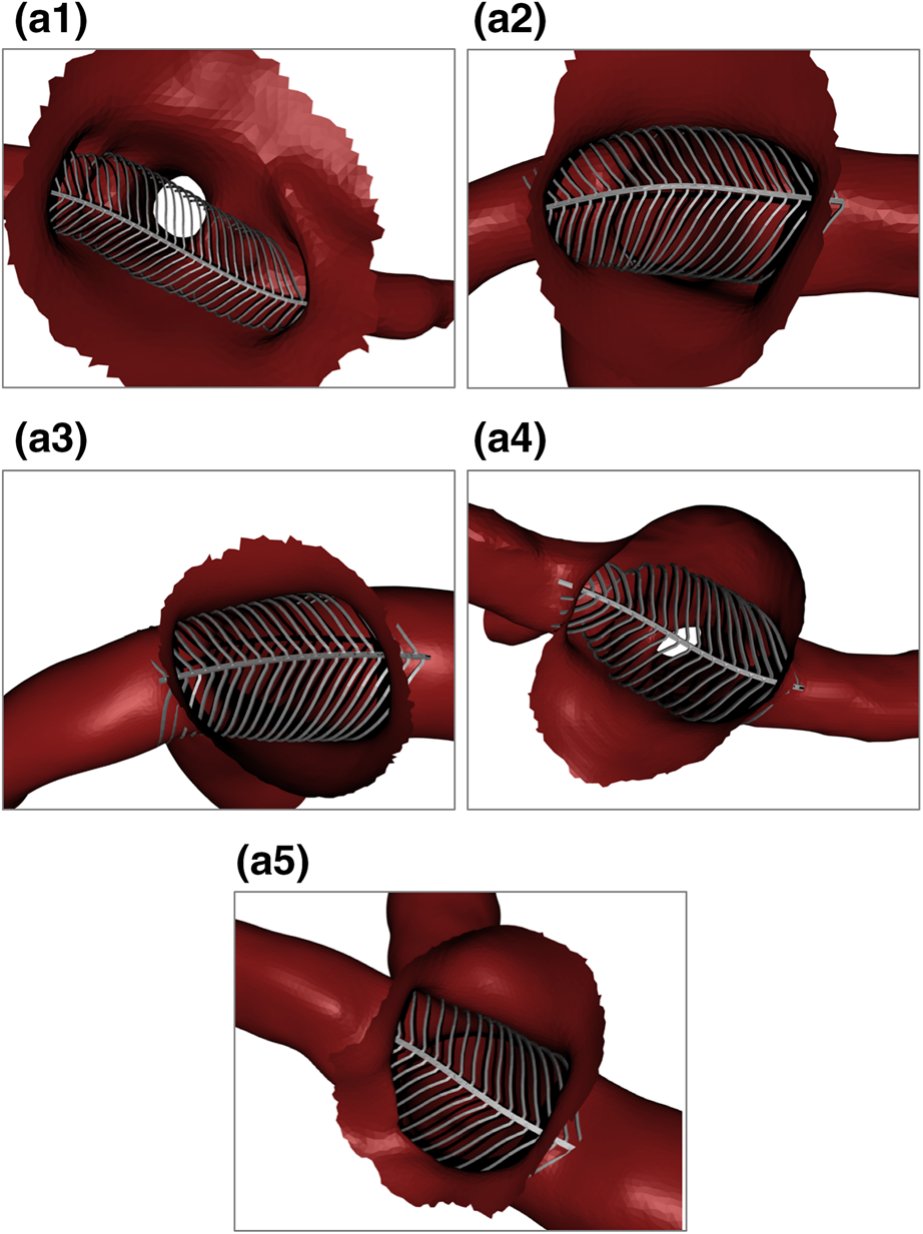
Deployed device configurations for each aneurysm geometry in Basilar tip (a1, a4) and Internal Carotid terminus (a2, a3, a5) locations. Aneurysm domes are partially removed for device inspection.

Five cerebral bifurcation aneurysm geometries (referred to as a1, a2, a3, a4, a5) were selected for virtual deployment and CFD simulation by the eVasc clinical team. Selection criteria for the geometries included appropriate aneurysm and vessel size to support treatment with the current generation of eCLIPs device (size range for daughter vessels 2.00–3.25 mm diameter). The selected geometries consisted of two basilar tip aneurysms (a1 and a4 in Fig. 2) and three internal carotid terminus aneurysms (a2, a3 and a5 in Fig. 2) with maximum aneurysm dome and neck diameters varying from 4.2 to 13.4 mm and 3.3 to 6.4 mm respectively.

For modelling purposes, the anchoring section of the device, shown in the right-hand portion of Fig. 1b, was ignored entirely as it was assumed to have no contribution towards aneurysm inflow reduction. It was also assumed that the anchoring portion is placed in the narrower/harder to access of the two daughter vessels, as per previous clinical use of the device.^29^

A simplified wireframe version of the device was deployed initially. The spine of the wireframe device was located across the aneurysm neck along a previously defined deployment line (Step I). The fully expanded wireframe device was then placed (Step II). All extraneous device ribs (those not covering the aneurysm neck in some way) were removed for computational efficiency. Each rib of the device was deformed into the approximate deployed configuration within the vessel (Step III). No material properties were assigned to the device and the local variation in rib stiffness due to cross-section or orientation was not incorporated in the wireframe. Instead, each wireframe rib was deformed with a pseudo-realistic condition of locally minimising deformed curvature in each rib, with the device spine remaining rigid. Each rib was trimmed after initial contact with the vessel wall to maximise computational efficiency. The wireframe device was then solidified to create ribs and a central spine of the same dimensions as the original device (Step IV). This procedure was used to deploy the same sized device in each of the five aneurysm geometries as shown in Fig. 2. Note that in large aneurysms with an amorphous neck (such as a1) the device can expand to its full equilibrium diameter and leave a gap between the device and vessel/aneurysm wall. In these cases the eCLIPs remains anchored in the substantially smaller daughter vessels.

### Mesh Independence

Case a2 was selected for a mesh independence study due to the relatively large flow velocity magnitude and gradient present in the aneurysm dome (identified in previous computations). The geometry with and without the eCLIPs device was meshed in CFD-VisCART (ESI Group) with a projected single domain unstructured mesh, an omnitree cartesian tree type and three near-wall cartesian layers to give a smooth and well-resolved boundary definition. This resulted in initial meshes of 298,000 and 85,000 cells respectively with and without the device deployed. The meshes were incrementally refined to give an approximate doubling of the element count, resulting in maximum mesh sizes of 24,300,000 and 14,100,000 cells respectively with seven levels of refinement.

For this test, steady state CFD computations were performed assuming a constant blood flow rate typical of the ICA (230 mL/min) and a radially symmetric, parabolic inlet velocity distribution. Blood was modelled with constant density and viscosity of 1000 kg/m^3^ and 0.004 Pa s respectively, resulting in a Reynolds number of 392 for this case. A plane was defined just within the aneurysm dome through which the aneurysm inflow was measured. The aneurysm dome surface was also isolated to measure WSS.

The reduction in aneurysm inflow due to the eCLIPs device at each level of mesh refinement was calculated and the expected convergence behaviour toward a fixed reduction with increased mesh refinement was confirmed. A mesh density greater than 5000 cells/mm^3^ was sufficient to capture the inflow reduction with less than 1% uncertainty. This finding is consistent with previous studies and devices.^21^,^23^ The corresponding reduction in mean and maximum wall shear stress (WSS) with increasing mesh fineness was also considered. In this case a level of mesh refinement greater than 5000 cells/mm^3^ was sufficient for mesh independence below 2%.

Overall a mesh fineness of at least 5000 cells/mm^3^ was chosen to provide sufficient confidence regarding mesh independence (1% on flow, 2% on shear stress) for all further computations.

### CFD Setup

Each aneurysm case with the eCLIPs device deployed was meshed to the previously defined level of mesh independence in CFD-VisCART (ESI Group), with the same meshing setup as for the mesh independence tests. Transient cardiac flow profiles corresponding to the ICA and BA were taken from a 1D model of the vascular network and scaled to achieve flow rates of 230 and 120 mL/min for the internal carotid and basilar arteries respectively.^27^ A constant heart rate of 75 BPM was assumed. Inlet velocity profiles were applied in a radially symmetric parabola consistent with Poiseuille flow, given the relatively low Womersley number (< 3). Constant pressure conditions were applied to all outlets with no substantial differences seen in relative proportions of outflow in each vessel when compared to *in vivo* observations in the literature.^12^,^30^ Vessel walls and the eCLIPs device were assumed rigid.^24^

Blood was modelled as an incompressible Newtonian fluid with a density of 1000 kg/m^3^ and a constant dynamic viscosity of 0.004 Pa s. The governing unsteady three-dimensional Navier–Stokes equations were solved using the finite volume approach in CFD-ACE + (ESI Group) employing a central differencing scheme (second order) for spatial interpolation and a Crank–Nicholson scheme (second order) for time marching. SIMPLE-Consistent (SIMPLEC) pressure correction was used in addition to an algebraic multi-grid for convergence acceleration.^20^,^34^,^35^ We have confirmed through sensitivity analysis that a constant time step of 0.001 s was sufficient and we used a convergence criterion of five orders of magnitude residual reduction or an absolute residual reduction to 1 × 10^−8^ to assert iterative convergence of all variables within each time step. Three full cardiac cycles were simulated (2.4 s real time) with results reported for the third cycle, to reduce transient effects. Mean Reynolds numbers in the range of 274 to 392 were observed across the five geometries, with an instantaneous peak of 980, which supported the assertion for the laminar nature of the flow.^10^,^14^ Small Womersley numbers (1.68–2.72) also confirmed that little departure of the velocity profile from the Poiseuille case occurred.^11^,^36^ Computations were run on 32–64 cores with 32–64 GB of RAM, depending on mesh size. Convergence was typically seen in fewer than 50 iterations per timestep and with a total solution time around 72–96 h.

### Post-processing

A plane was prescribed at each aneurysm neck through which inflow was measured. In the same spirit, the aneurysm dome was isolated from the parent vasculature for WSS computations. Post-processing was conducted in CFD-VIEW (ESI Group) and Matlab (Mathworks) yielding a number of flow metrics including aneurysm inflow (cycle averaged and cycle range), absolute intrasaccular velocity distributions, and both spatial mean and maximum TAWSS (Time-Averaged WSS). All time-averaged metrics were calculated from the flow distribution taken at 0.02 s intervals (40 sample points per cardiac cycle).

Reductions in these metrics (Inflow, velocities, TAWSSmean and TAWSSmax) are considered proxies to successful aneurysm treatment.^7^,^10^,^13^,^16^,^33^ Reduced inflow and lowered mean WSS are linked with thrombus initiation in the aneurysm dome, similarly a reduction in peak WSS is linked to reduced flow jetting and a reduction in rupture risk.^18^ However, the TAWSSmax metric can be misleading in the absence of a visualization of the shear stress distribution as the location of the peak WSS may change significantly after device deployment. An additional metric was calculated to capture the fraction of the aneurysm dome under shear stress conditions conducive to thrombosis initiation (a shear rate < 100 s^−1^ or WSS < 0.04 Pa s).^19^ This was quantified as the percentage area of the aneurysm dome under such conditions with and without a device deployed.

### Positioning Sensitivity Study

In order to quantify the effect of positioning uncertainty and variability on device performance, three further computations were conducted for Case a2. Specifically, the eCLIPs device was re-deployed in (1) the original position; (2) a “Reversed” configuration with the device anchored in the opposite daughter vessel; (3) a “Plus 15 Deg.” configuration with the original device rotated about its longitudinal axis by 15° and (4) a “Minus 15 Deg.” configuration with the original device rotated 15 degrees in the opposite direction. The same flow and shear stress metrics were computed for the three additional positions of the device in Case a2.

### Conventional Flow-Diverter Devices

The performance of the eCLIPs device was compared to a generic woven flow-diverter device (labelled as FD_BRAIDED_), similar to the SILK/SILK+ or Pipeline Embolization Device (PED). This device has a porosity of ~ 70% and was created in length and diameter combinations to mimic commercially available devices. The size of conventional device deployed in each aneurysm case is summarised in Table 1. Deployment and CFD modelling of these devices discussed in this paper were conducted previously with an identical meshing setup, boundary conditions and device sizing according to manufacturers’ recommendations. Details of these computations are referred to here for brevity and are available in the literature.^22^

**Table 1:** Size of conventional flow diverter device and eCLIPs device implanted into each aneurysm geometry

Aneurysm	Conventional device (FD_BRAIDED_) size	eCLIPs size
a1	3 × 25 mm	3.25 mm
a2	4 × 20 mm	3.25 mm
a3	4 × 20 mm	3.25 mm
a4	3 × 20 mm	3.25 mm
a5	3.5 × 20 mm	3.25 mm

## Results

**Figure 3:**
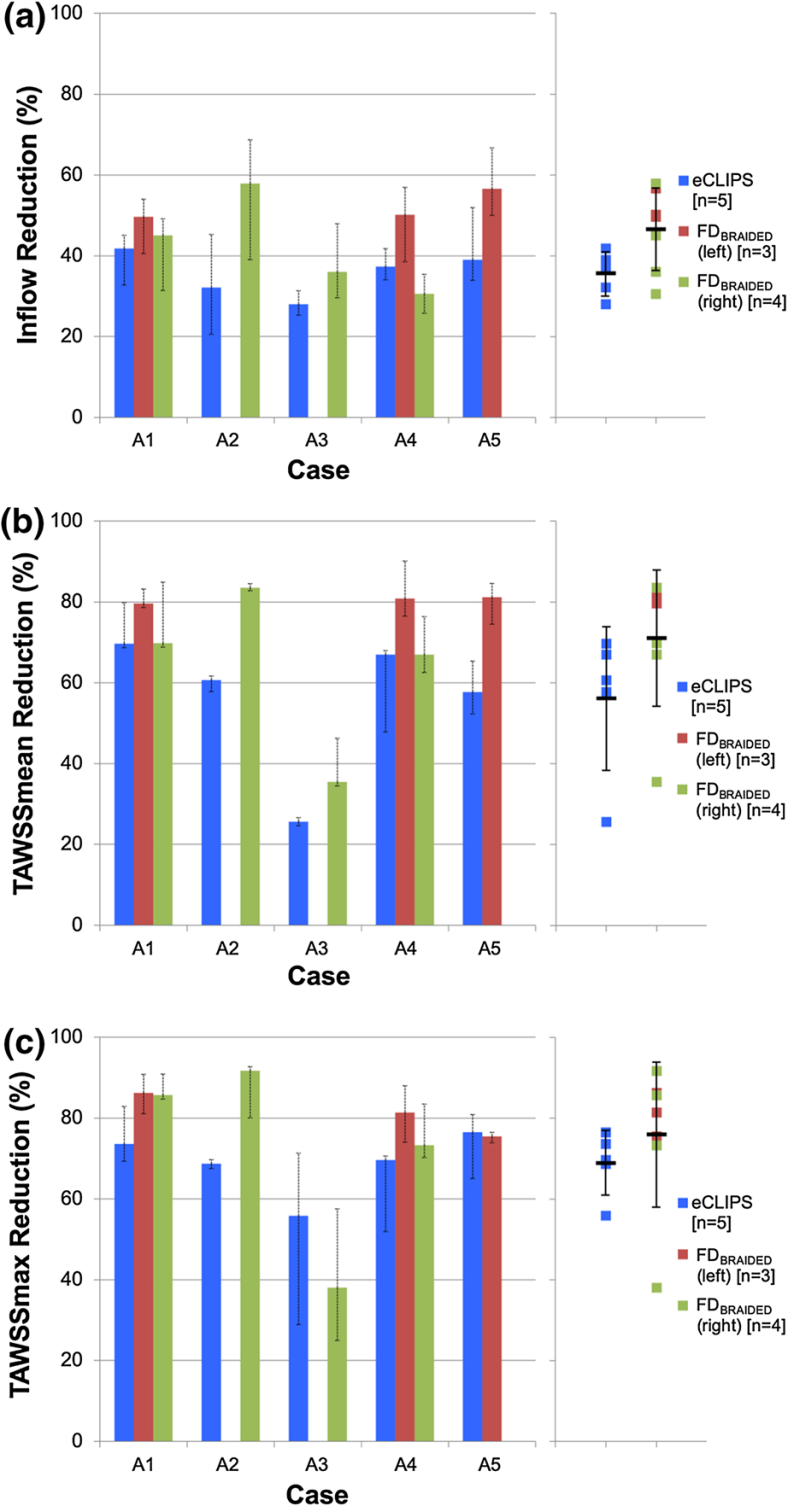
Reductions in inflow (a) and both mean (b) and maximum (c) TAWSS across the five aneurysm geometries due to the eCLIPs device and a conventional flow-diverter (FD_BRAIDED_). Columns and coloured markers both indicate cycle average, dashed error bars show cycle range. Solid black markers and solid error bars indicate mean and standard deviation of each distribution.

The cycle-averaged reductions in aneurysm Inflow, TAWSSmean and TAWSSmax due to the deployment of each device are plotted in Fig. 3 as both columns and coloured markers. The range of reductions seen across the cardiac cycle is indicated in dashed error bars for each column. In some geometries the conventional FD_BRAIDED_ device could be deployed in either daughter vessel under manufacturer recommendations and comparisons with both scenarios are shown. The plots on the right-hand side of Fig. 3 show a considerable spread of reductions about the mean and relatively large standard deviations for both devices, possibly excessively influenced by outliers in the low sample size. Across all three plots the lowest reductions seen correspond to Case G. Due to the low number of samples no tests for Normality, such as Shapiro-Wilks, could be performed. Consequently, the deviation of the data from Normal is unknown.

Across the five cases the eCLIPs device produces a relatively uniform and consistent reduction in aneurysm inflow of approximately 30–40%, perhaps due to the consistent size of the implanted device. The performance of the FD_BRAIDED_ on the other hand appears superior but more variable with inflow reductions between 30 and 60%, and a larger standard deviation in Fig. 3a. The reduction in mean TAWSS (TAWSSmean) is far more variable for both devices. In four treatment scenarios the FD_BRAIDED_ produces substantial reductions in TAWSSmean of around 80%, while the eCLIPs results in a reduction of around 60–70% in the same cases. This is reinforced by the larger standard deviation for both devices seen in Fig. 3b. A similar pattern emerges in the plot of reduction in peak TAWSS (TAWSSmax) shown in Fig. 3c with reductions in all cases, bar Case a3, of around 80% due to the FD_BRAIDED_ and 60–70% due to the eCLIPs. Across all three plots of Fig. 3 Case a3 stands out with both devices performing poorly and reducing both Inflow and TAWSSmean by around 30%.

**Figure 4:**
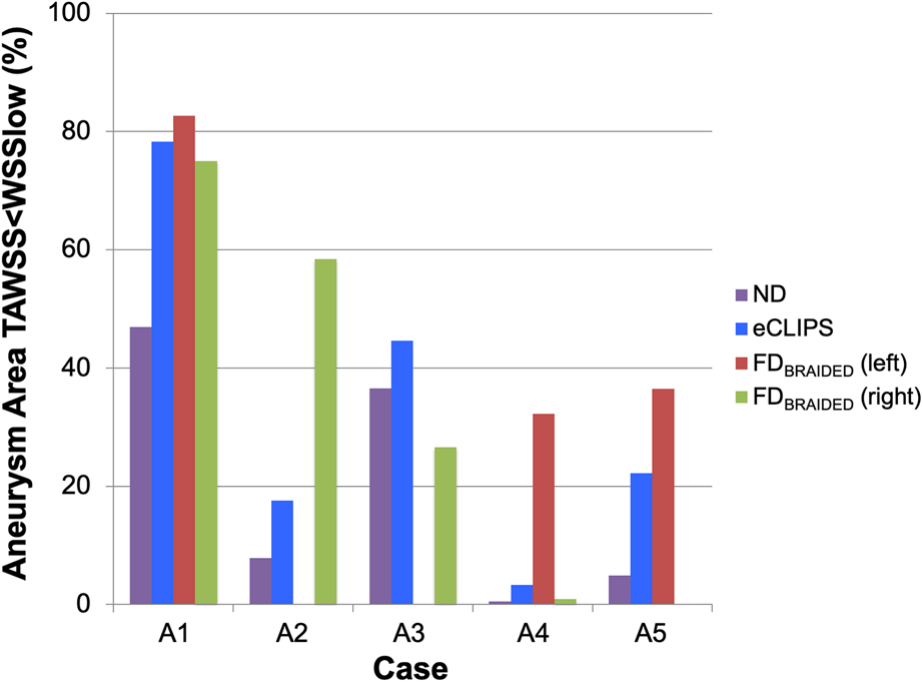
Aneurysm area below critical WSS for thrombus initiation displayed by case and device.

The corresponding area of the aneurysm dome with WSS magnitude that is low enough to initiate thrombosis is summarised in Fig. 4 for each device. In all combinations bar one (the FD_BRAIDED_ deployed in the right-hand vessel of Case a3) the presence of a device increases the area of the aneurysm dome under this preferential condition. Substantial variation in the aneurysm area fraction is seen across the cases and devices—ranging from around 1 to 80%. With the exception of Case a4 the FD_BRAIDED_ device increases this area to at least 30% across the cases regardless of deployment position. The eCLIPs device shows a larger range of aneurysm area fraction under preferential shear conditions—around 20–80% when case a4 is again excluded.

It should be noted that this area-based metric does not capture the risks associated with jets of flow on the aneurysm wall, which may only exist on a small area of the dome but have been linked with poor treatment outcomes. Similarly, the reduction in TAWSSmax may be misleading as the location at which the maximum TAWSS occurs is not considered; placement of a device could reduce a flow jet and lower TAWSSmax, but the resulting jet and TAWSSmax could impact a more critical area of the aneurysm, such as a vulnerable bleb. Detailed visual inspection of aneurysm flow and shear stress patterns is conducted in the next section.

Across the geometries daughter vessel outflow fractions were also compared for both the eCLIPs and conventional device. In both cases there was very little departure (< 5%) in outflow fraction compared to the No Device case suggesting that, in the first instance, neither device presents a daughter vessel occlusion risk. However, without incorporating biochemical models to capture device endothelialization the actual occlusion of the daughter vessel cannot be predicted.

**Figure 5:**
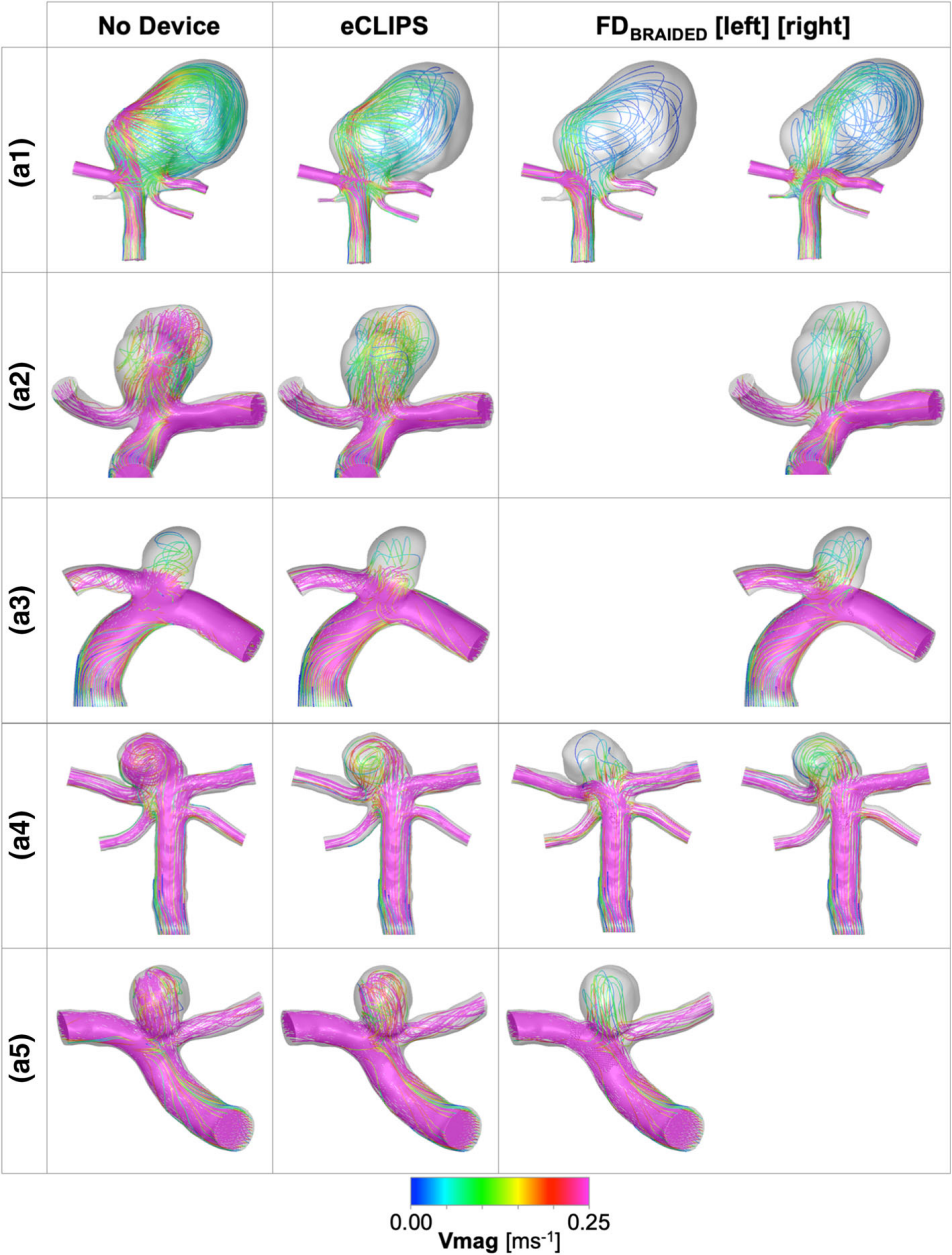
Velocity magnitude plots at mean flowrate for each aneurysm case a1–5 with and without a device deployed for both the eCLIPs and FD_BRAIDED_. Where the FD_BRAIDED_ has been deployed in either the left or right daughter vessel this is indicated.

**Figure 6:**
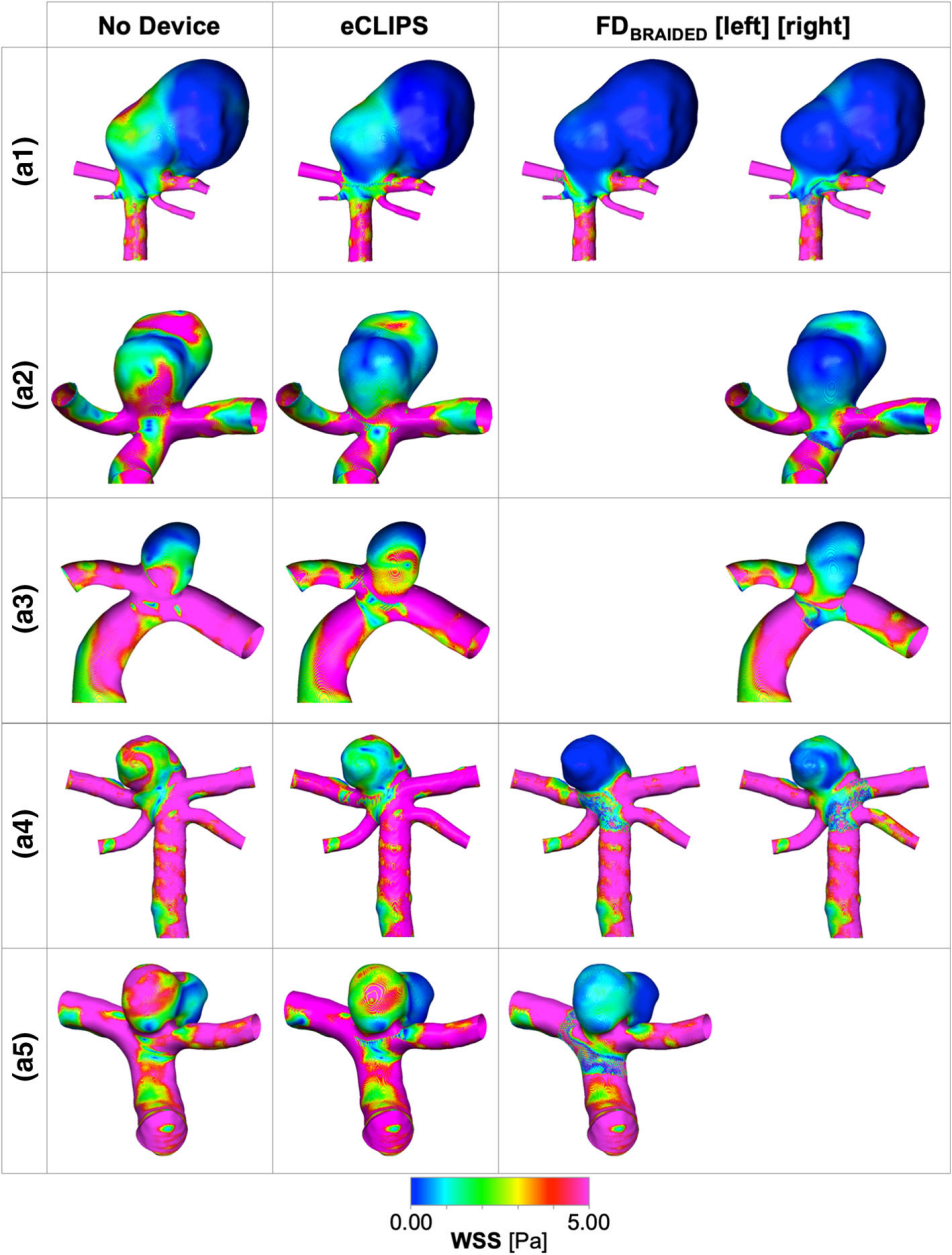
Wall shear stress (WSS) magnitude plots at mean flowrate for each aneurysm case a1–5 with and without a device deployed for both the eCLIPs and FD_BRAIDED_. Where the FD_BRAIDED_ has been deployed in either the left or right daughter vessel this is indicated.

Figures 5 and 6 show lines tangent to the instantaneous velocity vector, at mean flow rate, colour-coded for magnitude, and WSS distributions respectively at the same time step with and without eCLIPs or FD_BRAIDED_ devices.

### Case a1

In the a1 case, broadly similar performance is seen between the two device types and the two deployment configurations of the FD_BRAIDED_ device: inflow and WSS metrics are reduced by 40–50% and 70–80% respectively. The action of each device on the aneurysm flow pattern in a1 does vary: the conventional device diffuses the flow into a broader, less coherent jet that results in fewer areas of elevated WSS in the aneurysm dome when compared to the eCLIPs device. Overall the large size of the aneurysm dome in this case diffuses the relatively large inflow (even after device placement this averages ~ 60 mL/min) to create a shear stress environment conducive to thrombosis—reinforced by Fig. 4. The elimination of a jet-induced high WSS region by all devices is likely to address many of the clinical concerns for this large aneurysm.

### Case a2

Figures 5 and 6 summarise the same combination of velocity and WSS distributions for Case a2. In this case the eCLIPs device underperforms the FD_BRAIDED_ by around 20–30% when comparing reductions in key metrics. Across the velocity plots, it is clear that the fundamental aneurysm flow pattern is relatively unchanged by either device placement: flow enters at the back of the aneurysm and exits at the front, causing two peaks in WSS either side of the main aneurysm dome. These WSS peaks are reduced in severity and extent by both devices but more so by the smaller-pored FD_BRAIDED_, which reduces the entire aneurysm dome to a WSS below 2.5 Pa at mean flow. This is reflected in the plot of Fig. 4 where the conventional device places almost 60% of the aneurysm dome in a favourable shear stress environment, compared to the ~ 20% (approximately double the preferential area with no device present) achieved by the eCLIPs. In this case the relatively low reduction in aneurysm inflow for the eCLIPs device (32.2%) also translates into a high absolute inflow value after device placement, averaging ~ 70 mL/min—the highest post-device placement seen in this study. Together these factors would anecdotally point towards a reduced potential for aneurysm occlusion with the eCLIPs in this geometry.

### Case a3

Figures 5 and 6 summarises the equivalent distributions and metric reductions for Case a3. As was noted in Fig. 3, Case a3 shows markedly lower reductions in the three metrics considered for both devices (typically 30–40%), when compared to the other four aneurysm cases. However, these reductions should be considered alongside the relatively low absolute aneurysm inflow with or without a device, visible in the small number of flow lines entering the aneurysm dome. As such the aneurysm inflow rates seen after deployment of either device are the lowest across all five cases (~ 25 mL/min), which is likely low enough to encourage thrombosis. This point is reinforced by the more positive performance of Case a3 when considering the area of the aneurysm under WSS favourable for thrombosis (Fig. 4), but the same plot also shows the unusual result of the FD_BRAIDED_ device actually decreasing this area. This effect results from the FD_BRAIDED_ diffusing the concentrated flow entering the aneurysm into a slower moving core that then sweeps a larger fraction of the aneurysm dome, when compared to the more extreme high and low WSS areas visible in No Device and eCLIPs configurations.

The plot of TAWSSmax reduction in Fig. 3 is also somewhat misleading showing a greater reduction for the eCLIPs device when the FD_BRAIDED_ actually eliminates the WSS peak located at the aneurysm’s minor lobe. The poor performance of the conventional device captured in the graph has resulted from the higher WSS zone at the extreme left of the aneurysm dome (adjacent to the Anterior Communicating Artery) where flow leaving the aneurysm dome is concentrated.

Given the (pro-thrombotic) low aneurysm inflow rate, even in the absence of a device, the clinical concerns with this case are likely to focus on the high-WSS-inducing flow jet. Both the eCLIPs and FD_BRAIDED_ reduce the impact of this jet but the more effective jet reduction of the conventional device is offset by a corresponding increase in impingement of flow leaving the aneurysm. Such a concentrated aneurysm outflow region may correlate with the persistent neck remnants seen in the literature.^1^,^26^,^32^ There is also likely a secondary (positive) effect of the eCLIPs fully covering the aneurysm neck compared to the parent vessel placement of the FD_BRAIDED_ in that the eCLIPs presents a platform for endothelialization that spans the aneurysm neck entirely.

### Case a4

The velocity and WSS distributions for Case a4 are also shown in Figs. 5 and 6. In this case all devices reduce the violent flow pattern within the aneurysm dome and in particular the eCLIPs device and the conventional device deployed in the right-hand daughter vessel perform very similarly. Although the corresponding reductions in flow and shear stress metrics are similar between these two configurations, the FD_BRAIDED_ device again diffuses the flow jet entering the aneurysm more by reducing the velocity of the flow more substantially. This translates into a more effective reduction in shear stress peaks in the aneurysm dome with the FD_BRAIDED_ (right), which are still present with the eCLIPs device, but as with the previous case the area of peak WSS shifts to where flow exits the aneurysm in the FD_BRAIDED_ case.

Performance with the FD_BRAIDED_ device deployed in the opposite daughter vessel (left) is substantially improved. This better performance results from disrupting the fundamental flow pattern of the aneurysm dome, where flow now enters more centrally, and by the more tightly packed device struts restricting flow more—both effects documented in previous publications.^22^ This superior performance of the FD_BRAIDED_ deployed in the left vessel is very visible in Fig. 4 where all other device configurations only result in pro-thrombotic shear stress values in less than 5% of the aneurysm dome area.

The relatively high absolute inflow rates (~ 40–60 mL/min) in this small aneurysm with any device deployed, combined with the small fraction of aneurysm dome under pro-thrombotic shear stress (with the exception of the FD_BRAIDED_ left configuration) point towards low potential for aneurysm occlusion. It should also be noted that the superior performance of the conventional device results exclusively from the subtle choice of deployment location.

### Case a5

Finally, the flow distributions for Case a5 are shown in Figs. 5 and 6. For both the eCLIPs and FD_BRAIDED_ the presence of the device retains the “no device” flow pattern but reduces the speed and violence of the aneurysm flow. The shear stress patterns are correspondingly reduced in magnitude after device deployment but in both cases the fundamental WSS pattern of high and low locations remains. The eCLIPs underperforms the conventional device in inflow reduction by a substantial margin (~ 20%), which leads to similar differences in the reduction of mean TAWSS. As in previous cases this is visible as a more diffuse, low-velocity jet of flow entering the aneurysm in the conventional device case. Both devices also dramatically increase the fraction of aneurysm area under pro-thrombotic shear stress (Fig. 4) but again the FD_BRAIDED_ does so to a greater effect.

As with previous cases a less dramatic difference in TAWSSmax reduction is seen between the devices despite more effective jet elimination by the FD_BRAIDED_, and as previously, a corresponding increase in neck WSS is caused by exiting flow, which counters this reduction for the conventional device.

Large differences in absolute aneurysm inflow rate (~ 30 vs. ~ 45 mL/min) between the eCLIPs and FD_BRAIDED_ devices combined with more effective jet elimination and low shear stress promotion do point towards the FD_BRAIDED_ being the more effective treatment option for this case. However, the performance seen for the eCLIPs may also be sufficient for thrombosis and occlusion.

### Aneurysm Velocity Histograms

**Figure 7:**
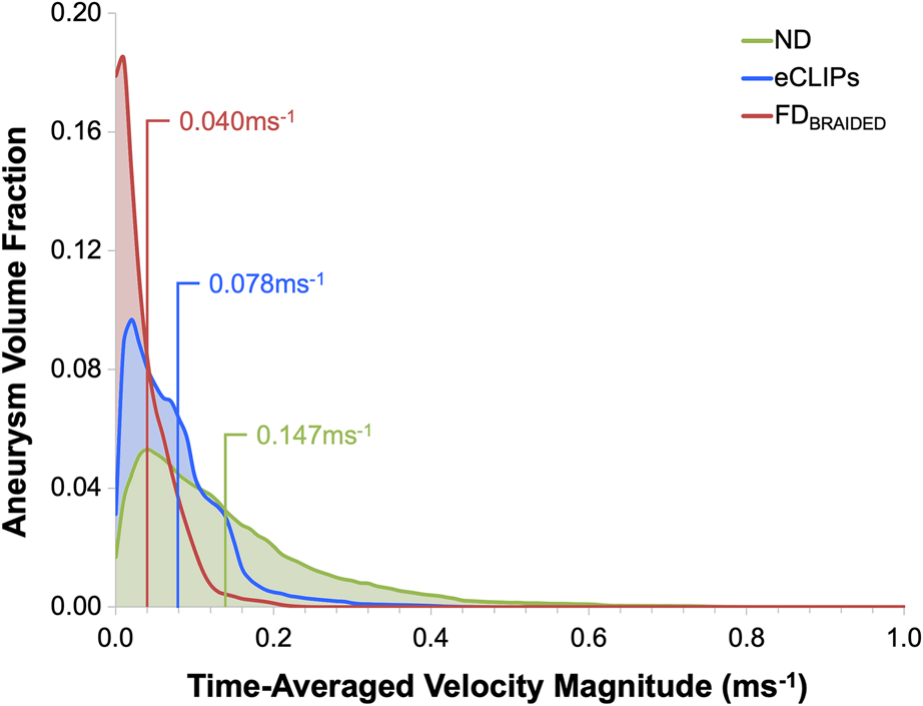
Aneurysm dome velocity histograms for Case a2 with and without devices deployed. Spatial means are indicated in corresponding coloured text.

Further detail of the flow within the aneurysm dome may be captured by considering the histogram of velocity magnitude within the aneurysm dome (J. Cebral 2018, personal communication), as shown for Case a2 in Fig. 7. Both the velocity histogram shape and mean value give an indication of the likelihood of thrombosis in the aneurysm dome—where stable thrombus is likely to be encouraged by a uniform low velocity throughout the aneurysm dome. The introduction of either the eCLIPs or FD_BRAIDED_ device in Case a2 reduces the mean dome velocity by 47 and 73%, respectively, while 90% of the flow in the aneurysm dome is below 0.310, 0.014, 0.08 ms^−1^ with No Device, the eCLIPs and FD_BRAIDED_ respectively. Equally the same flow distributions can be characterised by the fraction of the aneurysm dome with a velocity below 0.01 ms^−1^ as 7.0, 15 and 54% respectively for the No Device, eCLIPs and FD_BRAIDED_ configurations. These results broadly align with the inflow reduction seen for Case a2 where the eCLIPs produced an inflow reduction of approximately half that of the FD_BRAIDED_.

### Positioning Study Results

**Figure 8:**
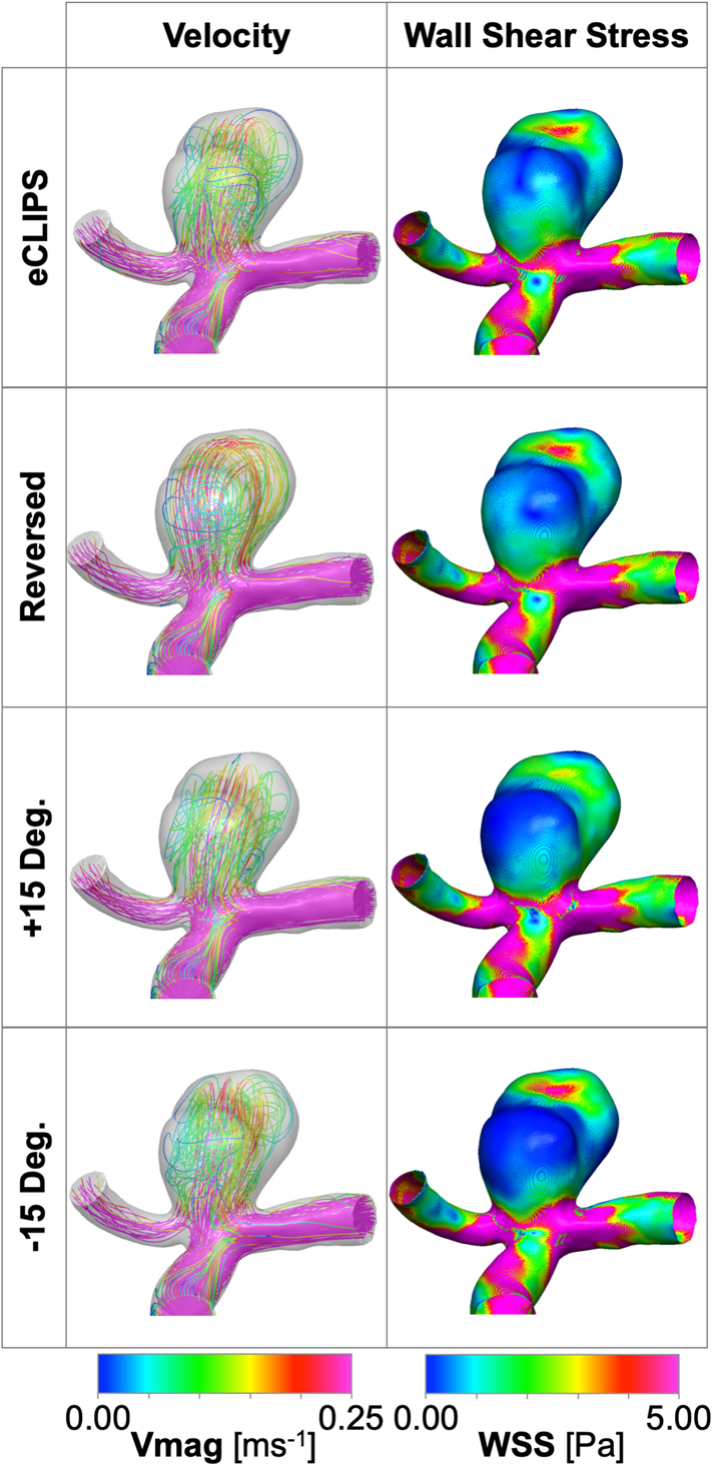
Case a2 positioning study results showing velocity and WSS distributions at mean flow rate with and without the eCLIPs and FD_BRAIDED_ devices deployed.

As shown in Fig. 8, the position of the eCLIPs device in a2 case does not appear to substantially affect the reductions in the three key metrics considered, with the exception of an almost 20% greater reduction in peak TAWSS when the device is reversed. The change in device position appears to mainly alter the location and number of high WSS areas in the aneurysm dome: two regions of approximately equal size and magnitude are present in the “eCLIPs” and “Minus 15 Deg.” configurations (visible on the front and back of the main aneurysm lobe) while a single region dominates in the “Reversed” and “Plus 15 Deg.” cases (a high WSS area on the front and back faces of the main aneurysm lobe). The superior TAWSSmax reduction performance of the “Reversed” device appears to correlate with the elimination of the high WSS region on the back face of the aneurysm dome. The “Reversed” configuration also produces a unique flow pattern: in all other configurations flow enters and exits the aneurysm centrally at the back and front respectively, whereas in the “Reversed” configuration flow enters on the left of the aneurysm dome towards the back face and exits on the right-hand side.

Similar results are seen for the area of aneurysm dome below a critical WSS. All positions of the device perform relatively poorly on this metric (20–30% area compared to > 50% for the FD_BRAIDED_) and again a slightly improved performance is seen in the reversed configuration where more of the aneurysm rear wall experiences lower TAWSS. Generally, the flow patterns and metric reductions appear relatively insensitive to variation in the positioning of the eCLIPs device.

## Study Limitations

Due to the speculative and computational modelling approach taken, this study has a necessary number of limitations. Primarily, only the instant after device implantation has been modelled—no capturing of the aneurysm thrombosis and device endothelialization that are necessary for successful treatment have been considered, only surrogate measures of these processes. Inferred boundary conditions of flow (population-averaged flowrate and parabolic profile) and outlet pressure (fixed) were implemented due to the lack of patient-specific data for the aneurysm cases concerned. The influence of these factors on the conclusions drawn in the study is considered small, given the like-for-like comparison with the known technology of a conventional flow diverter device.

## Conclusions

Overall the eCLIPs device exhibits a small but measurable performance reduction, when compared to a conventional flow-diverter (FD_BRAIDED_) deployed in the same five aneurysm cases. The eCLIPs typically reduced aneurysm inflow by around 10–20% less with similar reductions seen in mean shear stress. Small sample sizes mean it is not possible to interrogate the significance of this result.

On the other hand, the eCLIPs device consistently produced a uniform reduction in aneurysm inflow of ~ 30–40% under a wide range of aneurysm inflow rates prior to implantation (~ 40–115 mL/min). By contrast the FD_BRAIDED_ device produced flow reductions in the range of ~ 30–60%, where the consistency of the flow-diverter appears more dependent on deployed position and device sizing.

Compared to the conventional device the eCLIPs was less effective at diffusing jets of flow entering aneurysms and lowering the flow velocity. Consequently, local peaks in shear stress were less effectively reduced by the eCLIPs. This is likely due to the significantly larger effective pore size of the eCLIPs device and the lower pore density. This effect of pore size does not appear to adversely affect endothelialization, as previous pre-clinical studies of the eCLIPs have shown complete incorporation of the device 30–90 days after implantation.

The eCLIPs provided approximately equal resistance to flow entering and exiting the aneurysm dome—covering the entire neck—unlike the FD_BRAIDED_, which primarily restricted flow entering the aneurysm. In the case of the FD_BRAIDED_ this led to high-speed flow exiting the aneurysm in a very concentrated location in some cases, with a corresponding increase in local shear stress. This behaviour may correlate with the neck remnants recorded in the literature following FD treatment of bifurcation aneurysms.

These results confirm that the eCLIPs device can indeed act as a flow-diverter. The eCLIPs does not match the performance of conventional flow-diverters but it is unclear what degree of flow-diversion is sufficient for effective aneurysm isolation.
